# Application of a Monte Carlo‐based method for total scatter factors of small beams to new solid state micro‐detectors

**DOI:** 10.1120/jacmp.v10i1.2939

**Published:** 2009-01-27

**Authors:** Paolo Francescon, Stefania Cora, Carlo Cavedon, Paolo Scalchi

**Affiliations:** ^1^ Department of Medical Physics ULSS6 “Vicenza” Vicenza Italy

**Keywords:** small beams, total scatter factor, Monte Carlo, radiosurgery, Cyberknife

## Abstract

The scope of this work was to apply a method for estimation of total scatter factors of the smallest beams of the Cyberknife radiosurgery system to newly available solid‐state detectors: the PTW 60008 diode, the SunNuclear EdgeDetector™ diode, and the Thomson and Nielsen TN502RDM micromosfet. The method is based on a consistency check between Monte Carlo simulation of the detectors and experimental results, and was described in a recent publication. Corrected total scatter factors were in excellent agreement with the findings of the former study. The results showed that the diodes tend to overestimate the total scatter factor of small beams, probably due to excessive scatter from the material surrounding the active layer. The correction factor for diodes and for the micromosfet, however, was found to be independent of the electron beam width. This is a desirable characteristic because it allows standard correction factors to be used for treatment units of the same type, without the need of case‐by‐case Monte Carlo simulation.

PACS numbers: 87.55.kh; 87.55.ne; 87.56.Fc.

## I. INTRODUCTION

The scope of this work was to apply a recently published method for determination of total scatter factors ($s_{c,p}$) of the smallest beams of the Cyberknife radiosurgery system (Accuray, Inc., Sunnyvale, CA) by means of multiple detectors.^(1)^ A survey among users of the system revealed differences in $s_{c,p}$ values that are not consistent with actual differences between treatment units, thus calling for a method capable of accounting for effects that might alter the detector's response in very small fields. The method published in Francescon et al.^(1)^ provided details for two microchambers, a diode and a diamond detector, but did not include the objects of this study. These are the PTW 60008 diode, that had been considered less appropriate with respect to the 60012 model because of the presence of a metal filter,^(2)^ but nevertheless is widely used in clinical practice, and the SunNuclear EdgeDetector™ model 1118 diode, that became available only recently. The Thomson and Nielsen TN502RDM micromosfet was also included because of its small active volume compared to other solid‐state detectors and to the field size.

We believe that the method reported in Francescon et al.^(1)^ and briefly described below would be useful to institutions that do not have access to Monte Carlo resources or that prefer to avoid the whole simulation process. In fact, the simulated range of beam parameters should cover any existing treatment unit of the same type. However, the method requires that the details of the detectors used for measurement be simulated within the framework of the study, to make simulation results available to other users. This study was aimed at providing such simulation for the three new detectors.

## II. MATERIALS AND METHODS

The characteristics of the three detectors are summarized in Table 1.

**Table 1:** Characteristics of the detectors.

*DETECTOR*	*TYPE AND DIMENSION*	*MATERIAL*
PTW 60008	p‐type diode 1mm^2^ front area 2.5 μm thickness (depletion region)	Silicon, polyethylene, epoxy metal filter on backside
Sun Nuclear “Edge detector” mod. 1118	n‐type diode $0.8\times 0.8\;{\text{mm}}^{2}$ front area 2.5 μm thickness (depletion region)	Silicon, epoxy 0.13 mm‐thick brass housing of stem 2.3 mm aperture in front of the active layer 2.1 mm‐thick copper substrate
Thomson and Nielson TN502RDM micromosfet	mosfet detector $0.2\times 0.2\;{\text{mm}}^{2}$ front area 0.5 μm thickness (${\text{SiO}}_{2}$ layer)	${\text{SiO}}_{2}$, silicon, epoxy (bulb), polyimide

$S_{c,p}$ was defined as $D_{\text{coll}}/D_{60}$, where $D_{\text{coll}}$ was the dose measured with each collimator and $D_{60}$ was the reference dose measured with the 60 mm collimator. Measurements were performed in water with the effective point of measurement placed at 80 cm from the source and 1.5 cm depth (78.5 source to surface distance), corresponding to the point of maximum dose along the depth dose curve of the 60 mm collimator. The PTW 60008 diode was used with the stem parallel to the beam axis. The SunNuclear diode was used with the stem perpendicular to the beam axis. However due to the design of the detector, this orientation would expose the active layer equivalently to the PTW diode. The micromosfet was exposed both with the flat side and the round side (bulb) towards the radiation source, and results were averaged. The average deviation between the two orientations was 1.1% (max 2.4%).

The detectors were centered on the horizontal plane by means of a laser pointer, then their position was finely tuned (within ±0.2mm) to achieve the maximum signal intensity using the 5 mm collimator. All measurements were made with 50 monitor units and averaged over a series of at least 5 repeated runs. The standard deviation between repeated measurements was on average 0.9% (max 1.6%) for the micromosfet (within each orientation); the standard deviation was less than 0.1% for the diodes.

The method applied in this work and extended to the three detectors was described in Francescon et al.^(1)^ and is summarized below. It consists of using both simulation and experiment to assess total scatter factors of circular beams obtained by means of the 5 mm, 7.5 mm and 10 mm collimators. Simulations were performed in the hypothesis of three different values of beam energy (6.5, 7.0 and 7.5 MeV) and four values of the full width at half maximum (FWHM) of the electron beam incident on the target (1.4, 1.8, 2.2 and 2.6 mm). The specification provided by the manufacturer was FWHM in the range 1.5 mm – 2.0 mm. Energy was chosen first, based on tissue‐maximum ratios (TMR) data; the optimal value resulted to be 7.0 MeV.

**Figure 1:**
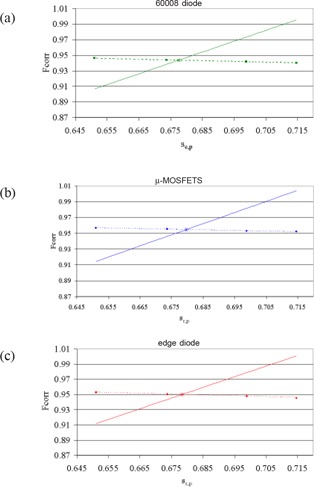
Graphical interpretation of the method based on a linear system. The the simulated values of $s_{c,p}$ and $F_{\text{corr}}$ in the hypothesis of the 4 different values of FWHM of the electron beam (dotted lines) and the result of the experimental measurement (continuous line). The abscissa of the point of intersection is the estimated $s_{c,p}^{*}$, while the ordinate is the estimated $F_{\text{corr}}$. PTW 60008 diode; the estimated $s_{c,p}^{*}$ value is 0.678. TN micromosfet; the estimated $s_{c,p}^{*}$ value is 0.680. SunNuclear “edge” diode; the estimated $s_{c,p}^{*}$ value is 0.678.

The FWHM of the electron beam was determined indirectly by solving a linear system whose variables are a correction factor to be applied to raw $s_{c,p}$ measurements for each detector ($F_{\text{corr}}$) and the estimated total scatter factor ($s_{c,p}$):

(1) $$\left\{ \begin{array}{l} F_{corr}=a\;s_{c,p}+b\\ s_{c,p}=F_{corr}\;s_{c,p}^{\;\;\;\;\;\;\;m} \end{array}\right.$$

where $s_{c,p}^{\;m}$ is the measured total scatter factor and a and b are the parameters of a linear fit between the four possible values of the pair ($F_{\text{corr}}$, $s_{c,p}$) corresponding to the four values of the FWHM. A graphical interpretation of the linear system above is given in Figure 1. For the calculation of the linear fit between the pairs, $s_{c,p}$ is the Monte Carlo‐calculated value in a small volume of water (i.e., without simulating the detector^(1)^) and $F_{\text{corr}}$ is the appropriate value taken from Table 2. Obviously, the solution to the linear system in terms of $F_{\text{corr}}$ and $s_{c,p}$ (denoted as $F_{\text{corr}}^{*}$ and $s_{c,p}^{*}$) already responds to the goal of the study. In the graphical representation of Figure 1, the abscissa of the point of intersection is the estimated $s_{c,p}^{*}$ while the ordinate is the estimated $F_{\text{corr}}^{*}$.

**Table 2:** $F_{\text{corr}}$ of the 3 detectors for the 5, 7.5, and 10 mm collimators (as a function of the FWHM).

*PTW 60008 FWHM (mm)*	*5mm coll*	$F_{\text{corr}}\;7.5\;\text{mm coll}$	*10 mm coll*
1.4	0.940	0.950	0.965
1.8	0.942	0.951	0.964
2.2	0.944	0.951	0.965
2.6	0.946	0.953	0.967

*TN mmosfet FWHM (mm)*	*5mm coll*	$F_{\text{corr}}\;7.5\;\text{mm coll}$	*10 mm coll*
1.4	0.952	0.989	0.999
1.8	0.953	0.990	1.000
2.2	0.955	0.991	1.000
2.6	0.957	0.992	0.999

*SunNuclear FWHM (mm)*	*5 mm coll*	$F_{\text{corr}}\;7.5\;\text{mm coll}$	*10 mm coll*
1.4	0.946	0.955	0.966
1.8	0.948	0.955	0.966
2.2	0.951	0.956	0.966
2.6	0.953	0.957	0.966

In this method, estimation of the FWHM is indirect, in other words it is a consequence rather than a prerequisite for simulation.

The absorbed dose in the active volume of the detectors embedded in a water phantom was simulated by means of CAVITY, an EGSnrc‐based code^(^ ^3^ ^,^ ^4^ ^)^ and the C++ class library, egspp, for use with the EGSnrc package, developed by Kawrakow.^(5)^ All simulations were performed until the achieved uncertainty was less than 0.15% (one standard deviation). The transport parameters were ECUT=512keV (total energy for electrons), PCUT=1keV (total energy for photons), ESTEPE=0.25, and XIMAX=0.5. The electron multiple scattering and boundary crossing algorithm was PRESTA‐II with the EXACT option. The composition of the detectors (whose details were provided by the manufacturers) has been simulated using PEGS4,^(3)^ with a cutoff energy of AE=512 keV for electrons and a photon energy cutoff, AP, of 1 keV. Density effect correction factors reported in ICRU Report 37^(6)^ have been used in the Monte Carlo model of the detectors. The active volume of the PTW diode was described as a 60 μm‐thick layer, while for the SunNuclear diode a 22μm layer was used.^(7)^ ^,^ ^(8)^. In fact, though the depletion region specified by the manufacturer is smaller (see Table 1), the active region is the diffusion length of the minority carriers (according to the studies reported in Rikner et al.^(7)^ and Shi et al.^(8)^). For the micromosfet, the active volume was identified with the 0.5 μm thick ${\text{SiO}}_{2}$ layer. Backscatter from the collimating system to the monitor chamber was not taken into account for all simulations. However, such effect was initially tested and found to be negligible (less than 0.5% on calculated $s_{c,p}$). The effect is lower than in conventional linear accelerators, probably due to the peculiar geometry of the collimating system, the smaller volume of the monitor chambers, and the smaller reference field (28 cm^2^ circular versus 100 cm^2^ square).^(9)^

## III. RESULTS

Table 2 shows the values of $F_{\text{corr}}$ to be applied to raw measurements of $s_{c,p}$ as a function of the FWHM of the electron beam. This Table should be used to estimate $F_{\text{corr}}^{*}$ and $s_{c,p}^{*}$ for a treatment unit different from the one investigated in this study, according to the flowchart in Francescon et al.^(1)^

In other words, and to better explain the method, $F_{\text{corr}}$ in Table 2 is the ratio between the “supposed true” $s_{c,p}$ (simulated in water) and the expected $s_{c,p}$ (simulated in the active volume of the actual detector). In this sense, $F_{\text{corr}}$ is thus already determined by simulation without need of measurement; however, $F_{\text{corr}}$ is a function of the electron beam width (FWHM). The role of the measured $s_{c,p}$ is to compare with the simulated detector response (through linear system^(1)^) in order to pick up the correct value of beam width, thus univocally determining the $F_{\text{corr}}$ value and the “supposed true” $s_{c,p}$.

The estimated $F_{\text{corr}}^{*}$ and $s_{c,p}^{*}$ for the three solid state detectors are reported in Table 3.

**Table 3:** Estimated $F_{\text{corr}}^{*}$ and $s_{c,p}^{*}$ for the 5 mm, 7.5 mm, and 10 mm collimators for the 3 detectors simulated in this study.

	5 mm		7.5 mm		10 mm	
	$F_{\text{corr}}^{*}$	$s_{c,p}^{*}$	$F_{\text{corr}}^{*}$	$s_{c,p}^{*}$	$F_{\text{corr}}^{*}$	$s_{c,p}^{*}$
PTW 60008	0.944	0.678	09.50	0.825	0.965	0.876
TN μmosfet	0.955	0.680	0.991	0.817	1.000	0.872
SunNuclear	0.950	0.678	0.955	0.823	0.966	0.877
mean $s_{c,p}$		0.679		0.822		0.875
±2σ		±0.002		±0.008		±0.006

**Table 4:** Measured and MC‐simulated $s_{c,p}$, for the 3 detectors and for the 5, 7.5, and 10 mm collimators for the various FWHM of the Gaussian spatial distribution of the electron source.

	*measured* $s_{c,p}$	*FWHM 1.4 mm simulated* $s_{c,p}$	*FWHM 1.8 mm simulated* $s_{c,p}$	*FWHM 2.2 mm simulated* $s_{c,p}$	*FWHM 2.6 mm simulated* $s_{c,p}$
Coll 5 mm					
PTW 60008	0.718	0.760	0.742	0.714	0.688
TN μmosfet	0.712	0.751	0.733	0.706	0.680
SunNuclear	0.714	0.756	0.737	0.711	0.680
Coll 7.5 mm PTW 60008	0.868	0.869	0.864	0.857	0.854
TN μmosfet	0.825	0.835	0.830	0.822	0.820
SunNuclear	0.862	0.865	0.859	0.852	0.850
Coll 10 mm PTW 60008	0.908	0.914	0.909	0.902	0.896
TN μmosfet	0.872	0.882	0.876	0.871	0.867
SunNuclear	0.908	0.913	0.907	0.902	0.897

Figure 1 shows a graphical interpretation of the linear system whose solutions are the values given in Table 3. Only the data relative to the 5 mm collimator are reported. The estimated $s_{c,p}^{*}$ values resulting from solution of the linear system are 0.678, 0.680 and 0.678 for the PTW 60008 diode, the TN micromosfet and the SunNuclear “edge” diode. Raw measurements gave 0.718, 0.712 and 0.714, respectively (see also Table 4).

Table 4 reports measured and MC‐simulated $s_{c,p}$ (that is, the *expected measured value* as a result of the Monte Carlo simulation of the detector) for the 3 detectors and for the 5, 7.5, and 10 mm collimators, in the hypothesis of the four different values of the FWHM of the Gaussian distribution of the electron source.

## IV. DISCUSSION & CONCLUSIONS

The results of this study confirmed some findings obtained in previous investigations.

When compared with Francescon et al.^(1)^, the estimated values of $s_{c,p}$ in this study were on average 0.679±0.002 versus 0.677±0.004 for the 5 mm collimator, 0.822±0.008 versus 0.820±0.008 for the 7.5 mm collimator, and 0.875±0.006 versus 0.871±0.008 for the 10 mm collimator.

The correction factors for small solid state detectors were found to be lower than 1, which is consistent with previous studies and with the hypothesis of excessive scatter reaching the active layer due to the relatively high atomic number of the silicon substrate (and metal filters, if present) compared to water.

The study reported in Francescon et al. included one diode detector, for which the variation of $F_{\text{corr}}$ was found to be practically independent of the FWHM of the electron beam. The study reported here confirmed this behaviour for two different diodes, a desirable characteristic that allows users to apply a standard correction factor to the measured $s_{c,p}$ independently of fine tuning details of the treatment unit. This feature is shared by the TN micromosfet which, however, is affected by higher statistical uncertainty. On the other hand, correction factors for the micromosfet are closer to 1 compared to diodes – apart for the smallest field (5 mm) for which even a small detector like the micromosfet produces a non‐negligible perturbation. The microchambers and diamond detector previously investigated did not show independence of the correction factor from the size of the electron source, thus requiring a more complicated approach to the correction of experimental results.
